# Genomic Diversity of the Ostreid Herpesvirus Type 1 Across Time and Location and Among Host Species

**DOI:** 10.3389/fmicb.2021.711377

**Published:** 2021-07-13

**Authors:** Benjamin Morga, Maude Jacquot, Camille Pelletier, Germain Chevignon, Lionel Dégremont, Antoine Biétry, Jean-François Pepin, Serge Heurtebise, Jean-Michel Escoubas, Tim P. Bean, Umberto Rosani, Chang-Ming Bai, Tristan Renault, Jean-Baptiste Lamy

**Affiliations:** ^1^Ifremer, RBE-SGMM-LGPMM, La Tremblade, France; ^2^Ifremer, ODE-Littoral-Laboratoire Environnement Ressources des Pertuis Charentais (LER-PC), La Tremblade, France; ^3^IHPE, CNRS, Ifremer, Université de Montpellier – Université de Perpignan Via Domitia, Montpellier, France; ^4^The Roslin Institute and Royal (Dick) School of Veterinary Studies, University of Edinburgh, Midlothian, United Kingdom; ^5^Centre for Environment, Fisheries and Aquaculture Science, Weymouth, United Kingdom; ^6^Department of Biology, University of Padua, Padua, Italy; ^7^Yellow Sea Fisheries Research Institute, CAFS, Qingdao, China; ^8^Ifremer, RBE, Nantes, France

**Keywords:** marine virus, OsHV-1, diversity, shellfish farming, measurably evolution

## Abstract

The mechanisms underlying virus emergence are rarely well understood, making the appearance of outbreaks largely unpredictable. This is particularly true for pathogens with low per-site mutation rates, such as DNA viruses, that do not exhibit a large amount of evolutionary change among genetic sequences sampled at different time points. However, whole-genome sequencing can reveal the accumulation of novel genetic variation between samples, promising to render most, if not all, microbial pathogens measurably evolving and suitable for analytical techniques derived from population genetic theory. Here, we aim to assess the measurability of evolution on epidemiological time scales of the Ostreid herpesvirus 1 (OsHV-1), a double stranded DNA virus of which a new variant, OsHV-1 μVar, emerged in France in 2008, spreading across Europe and causing dramatic economic and ecological damage. We performed phylogenetic analyses of heterochronous (*n* = 21) OsHV-1 genomes sampled worldwide. Results show sufficient temporal signal in the viral sequences to proceed with phylogenetic molecular clock analyses and they indicate that the genetic diversity seen in these OsHV-1 isolates has arisen within the past three decades. OsHV-1 samples from France and New Zealand did not cluster together suggesting a spatial structuration of the viral populations. The genome-wide study of simple and complex polymorphisms shows that specific genomic regions are deleted in several isolates or accumulate a high number of substitutions. These contrasting and non-random patterns of polymorphism suggest that some genomic regions are affected by strong selective pressures. Interestingly, we also found variant genotypes within all infected individuals. Altogether, these results provide baseline evidence that whole genome sequencing could be used to study population dynamic processes of OsHV-1, and more broadly herpesviruses.

## Introduction

A common paradigm is that double-stranded DNA (dsDNA) viruses, and particularly herpesviruses, are inherently stable (Sanjuán et al., 2010; Sanjuán and Domingo-Calap, 2016) making them unsuitable for analytical techniques derived from population genetic theory. At the molecular level, this view stems from low per-site mutation rates due to the high-fidelity polymerases and error correction systems typical of these viruses. However, recent progress has been made in understanding the genomic diversity and evolution of mainly human herpesviruses, driven by the rapid expansion and application of high throughput sequencing (HTS), bioinformatics, and comparative genomics in virology. The ability of dsDNA viruses to accrue standing variation, and to undergo recombination with neighboring genomes, creates many opportunities for selective pressures to induce rapid genetic shifts (for a review of factors see Sanjuán and Domingo-Calap, 2016). In the meantime, estimates of the substitution rates of human herpesviruses and cytomegaloviruses are substantially higher than previously though (Kitchen et al., 2010; Jaramillo et al., 2013; Parsons et al., 2015; Renner and Szpara, 2017) and evidences that several viral haplotypes can be found within one infected host (at the individual scale) have been provided (Renzette et al., 2011; Cudini et al., 2019). Together, these data have reshaped our sense of the stability of herpesvirus genomes. Because contemporary viral genomic diversity is a result of the dynamic interaction of past ecological and evolutionary processes (Retel et al., 2019), opportunities to assess their evolution, at a genome-wide scale, to infer their population dynamics from genomic data (Biek et al., 2015) offer new perspectives for better understanding and preventing herpesviruses outbreaks.

Ostreid herpesvirus 1 (OsHV-1) is a dsDNA herpesvirus and the unique member of the genus Ostreavirus (family Malacoherpesviridae, order Herpesvirales). It was the first herpesvirus isolated from invertebrates in the early 1990s (Nicolas et al., 1992) and likely is the causative agent of the last decades increased mortality events in Pacific oysters, *Crassostrea gigas* and therefore responsible for dramatic economic damages (EFSA, 2010; Segarra et al., 2010). Since the appearance of the OsHV-1 μVar variant in 2008, we have observed high mortality following the detection of this variant, the means to fight against the infection of this virus in oyster are limited.

Ostreid herpesvirus 1 infects mainly spat and juvenile *C*. *gigas* and is currently detected in most oyster producing area worldwide (Nicolas et al., 1992; Garcia et al., 2011; Paul-Pont et al., 2013). Furthermore, this virus is able to infect at least four more distantly related marine bivalve species (*Ostrea edulis*, *Chlamys farreri*, *Pecten maximus Cerastoderma edule*, *and Anadara broughtonii*; Arzul et al., 2001, 2017; Davison et al., 2005; Bai et al., 2015; Bookelaar et al., 2020). However, whether viral populations infecting the different host species are the same remains unknown.

The first variant of OsHV-1 was described on *P*. *maximus* larvae and was named OsHV-1 Var but the associated disease was not well documented (Arzul et al., 2001). Since 2008, microvariants of OsHV-1 have been associated with increasing mortality of *C*. *gig*as in France, and in a number of countries across Europe, as well as in Australia and New Zealand (Segarra et al., 2010; Jenkins et al., 2013). OsHV-1 μVar is a single variant which differs from the reference genome published in 2005 by Davidson (accession number NC_005881.2) mainly because of sequence variations in a microsatellite locus upstream of the Open Reading Frame (ORF) 4, in ORF4 and in ORF42/43. The term “microvariants” is now used to refer to all haplotypes with mutations in and upstream of ORF 4 and in ORF 42/43. Other variants have also been described, for example OsHV-1-SB which has been associated with mass mortalities of *S*. *broughtonii* in China (Bai et al., 2015, 2016).

Following the emergence of OsHV-1 μVar, a number of studies have been undertaken to investigate the diversity of OsHV-1 using single- (Renault et al., 2014) or multi-genomic regions (Renault et al., 2012). Three OsHV-1 genome sequences were obtained with bacteriophage lambda libraries or genome walking procedures (Davison et al., 2005; Ren et al., 2013; Xia et al., 2015). More recently, three additional OsHV-1 μVar genomes were sequenced using Illumina shotgun DNA-seq technology (Burioli et al., 2017; Abbadi et al., 2018).

While sequencing allows us to characterize polymorphism and genomic variation at the genome scale with unprecedented power, in the case of OsHV-1, diversity has not yet been explored at the genome scale, or across different dimensions such as time, location and host species. Recently a study of viral diversity was performed at the viral mRNA level, which demonstrated viral populations differ depending on the genetic background of their hosts. This result suggests a co-evolution process between OsHV-1 μVar and oyster populations (Delmotte et al., 2020).

Therefore, using a reference mapping approach from historical collection of deep sequenced infected animals, we aim to assess the measurability of evolution on epidemiological time scales of OsHV-1. We also aim at characterizing the potential of the approach to test (i) if infected individuals belonging to different host species harbor different viral populations with large genomic differences possibly due to viral speciation and, (ii) if infected individual from the same host species but originated from different world region harbor viral populations with the same genomic structure and simple polymorphisms.

## Materials and Methods

### Sample Collection

Samples analyzed in this study come from a combination of sources, including an historical collection and the European and National Reference Laboratory for Bivalve Mollusc Diseases. For the rest of this paper, we refer to “*isolates*” in the sense of viral populations isolated from infected individual host. Most of the data derives from whole-genome sequencing of infected individual host or pools of infected individual hosts (Table 1). The 22 isolates in this study were collected from several countries around the world: France (eight infected juveniles *C*. *gigas*, two pool of infected *C*. *gigas* oysters, and one *C*. *gigas* adult), New-Zealand (one infected pool of *C*. *gigas* larvae and three infected *C*. *gigas* adults), Ireland (one *C*. *gigas* infected adult), Japan (two *C*. *gigas* infected adults), Sweden (two pooled infected *O*. *edulis* larvae and one infected adult), United-Kingdom (one *C*. *gigas* individual), Netherlands (one *Crassostrea gigas* infected adult), and Spain (one *C*. *gigas* infected adult; Table 1).

**TABLE 1:** Description of the 22 sequenced libraries in this study: country of origin, host species, year of collection, sample stage, sequencing technology, and number of reads aligned against Ostreid herpesvirus 1 (*OsHV-1*) reference genome (Accession number: NC_005881.2).

Sample’s ID	Country of origin	Species	Year	Stage	Sequencer, sequencing protocol, platform	Aligned reads
VIV46-2-m	France	*Crassostrea gigas*	2017	Juvenile	HiSeq 4000, Paired-end 150 bp, LIGAN-PM Genomics	281,336
VIV56-10-m	France	*Crassostrea gigas*	2017	Juvenile	HiSeq 4000, Paired-end 150 bp, LIGAN-PM Genomics	346,491
VIV48-4-m	France	*Crassostrea gigas*	2017	Juvenile	HiSeq 4000, Paired-end 150 bp, LIGAN-PM Genomics	354836
VIV49-5-m88	France	*Crassostrea gigas*	2017	Juvenile	HiSeq 4000, Paired-end 150 bp, LIGAN-PM Genomics	183,665
VIV58-12-m	France	*Crassostrea gigas*	2017	Juvenile	HiSeq 4000, Paired-end 150 bp, LIGAN-PM Genomics	155,976
VIV47-3-m	France	*Crassostrea gigas*	2017	Juvenile	HiSeq 4000, Paired-end 150 bp, LIGAN-PM Genomics	370,770
VIV57-11-m99	France	*Crassostrea gigas*	2017	Juvenile	HiSeq 4000, Paired-end 150 bp, LIGAN-PM Genomics	49,985
Poole-Harbour United Kingdom	United-Kingdom	*Crassostrea gigas*	2015	na	MiSeq, Paired-end 150 bp	55,056
LI	France	*Crassostrea gigas*	2010	Pool of larvae	HiSeq 2500, Paired-end 100 bp, GenoToul	400,021
PR	France	*Crassostrea gigas*	2008	Pool of juveniles	HiSeq 2500, Single-end 100 bp, GATC Biotech	344,852
MV	France	*Crassostrea gigas*	2010	Ripe Adult	HiSeq 2500, Single-end 100 bp, GATC Biotech	331,278
NZ	New-Zealand	*Crassostrea gigas*	2010	Pool of larvae	HiSeq 2500, Single-end 100 bp, GATC Biotech	341256
NZ16	New-Zealand	*Crassostrea gigas*	2011	Adult	HiSeq 2500, Paired-end 100 bp, GATC Biotech	105,610
NZ17	New-Zealand	*Crassostrea* gigas	2011	Adult	HiSeq 2500, Paired-end 100 bp, GenoToul	202,246
NZ18	New-Zealand	*Crassostrea gigas*	2011	Adult	HiSeq 2500, Paired-end 100 bp, GenoToul	150,315
IRL15*	Ireland	*Crassostrea gigas*	2011	Adult	HiSeq 2500, Paired-end 100 bp, GenoToul	1,802
JP2*	Japan	*Crassostrea gigas*	na	Adult	HiSeq 2500, Paired-end 100 bp, GenoToul	2,115
JP6*	Japan	*Crassostrea gigas*	na	Adult	HiSeq 2500, Paired-end 100 bp, GenoToul	3,280
NL4*	Netherlands	*Crassostrea gigas*	na	Adult	HiSeq 2500, Paired-end 100 bp, GenoToul	4,143
SP16*	Spain	*Crassostrea gigas*	na	Adult	HiSeq 2500, Paired-end 100 bp, GenoToul	1,259
SW3*	Sweden	*Ostrea edulis*	2012	Pool of larvae	HiSeq 2500, Paired-end 100 bp, GenoToul	1,839
SW6	Sweden	*Ostrea edulis*	2012	Adult	HiSeq 2500, Paired-end 100 bp, GenoToul	402,082

Samples for which the ID starts by “VIV” are from research project Vivaldi and the remaining samples are from historical collections. *Those samples did not pass the quality check and were discarded.

The historical samples were from moribund oysters collected during mortality outbreaks. For spat, juvenile and adults, a piece of mantle or gill was collected and stored in 90% ethanol until DNA extraction. For larvae samples, a pool of individuals was frozen (−20°C) and stored until DNA extraction.

All “VIV” oysters were sampled during a mortality outbreak in July 2017 (Table 1). Briefly, spat were placed into the field (*17390 La Tremblade Ronce-les-Bains France*, *a sandbank called* “*La Floride: LAT 45*.*80*° and *LONG -1*.*15*°). As soon as the onset of the mortality, spat were transferred in the laboratory and checked twice a day (from 18/07/2017 to 21/07/2017) to sample fresh moribund individuals.

### Genomic Library Preparation and Sequencing Runs

A range of 60–200 mg of pooled larval samples were mechanically crushed using unique piston pellet before DNA extraction. About 50 mg of fresh tissue were collected from spat-juveniles-adults. Nucleic acid extraction was performed using the QIAamp DNA Mini Kit (Qiagen) according to the manufacturer’s handbook. All extracted DNA samples were stored at −25°C to avoid any degradation and DNA concentrations were measured with the Nanodrop^TM^ (Labtech, France). After storage, DNA quality was assessed with Nanodrop^TM^ and checked again by the sequencing platform using fluorometric measurement of nucleic acids.

All the libraries were sequenced using Illumina technology, with HiSeq 2000 sequencers on two platforms, GATC (Mulhouse, France) and GenoToul (Toulouse, France), with the exception of United Kingdom samples that were sequenced with MiSeq on in-house sequencers, and the HiSeq 4000 for the most recent samples on LIGAN (CNRS, Lille, France). Regarding the sequencing protocols, three libraries were sequenced on single-end protocol (1 × 100 bp) and remaining libraries were sequenced with paired-end protocol (2 × 150 bp) (Table 1), with the exception of the Miseq data, which was generated using V3-600 Miseq specific cartridge. Raw sequence data are available on the NCBI Sequence Read Archive (SRA) under the BioProject ID: PRJNA721248.

### Bioinformatics and Genomic Framework

#### Read Mapping and Filtration

**FIGURE 1:**
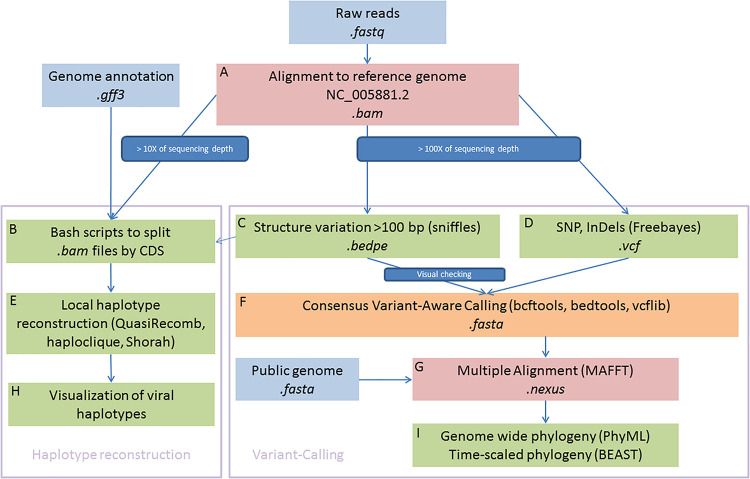
Schematic illustration of bioinformatics framework. In summary, the *right part* is devoted to genome-wide phylogenetic approaches and the *left part* aims to reconstruct local viral haplotypes. Sequences were aligned to reference genome **(A)** and consensus were used to detected haplotypes **(B,E,H)** and SNPs **(C,D,F)**. Multiple alignement were used to built phylogenies **(G,I)**.

A reference mapping approach was used to explore the viral genomic diversity within and between samples. This approach has been extensively used for eukaryotic organisms and is best suited to call single nucleotide polymorphisms (SNPs) and genomic structural variation (SV) from a collection of individuals or pooled libraries in comparison to the more widely used *de novo* assembly strategies in the viral community (Baaijens et al., 2017). All sequenced libraries were aligned against the OsHV-1 reference genome (Accession number: NC_005881.2) (Davison et al., 2005) using BWA MEM v7.1.0 (Li and Durbin, 2009; Li, 2013). Only samples with a mean of coverage depth equal to or above 100X have been included for polymorphism calling and 10X for haplotype reconstruction (Figure 1A).

#### Local Haplotypes Reconstruction

We first aimed to reconstruct local haplotypes. As far as we are aware, no software dedicated to haplotype reconstruction is able to manage long indels, as it is observed in OsHV-1 genomes, with short reads (i.e., ∼100 bp reads long). To circumvent this limitation, we decided to make local haplotype reconstruction on each gene coding DNA sequence (CDS) that were present in every sample and outside of inverted and repeat regions. Each CDS coordinate were extracted from the GenBank file (Clark et al., 2016) and we use them to subset the bam file for each sample (Locus_tag, Figure 1B). We feed QuasiRecomb (Töpfer et al., 2013), haploclique (Töpfer et al., 2014), and shorah (Zagordi et al., 2010, 2011) with CDS-split bam files (Figure 1E). Only results from Shorah software are showed on the main text because it represents the most conservative short read local assembler, whereas the other software outputs are showed in supplementary figures. Briefly, Shorah employs a model-based probabilistic clustering algorithm to correct errors, infer haplotypes and their frequencies, and estimate, using a Bayesian approach, the quality of the reconstruction by computing the full joint posterior probability distribution of all parameters of interest (Zagordi et al., 2010).

Subsequent plotting were made in R Development Core Team (2008) with basics functions and the ggplot2 package (Wickham, 2016) (Figure 1H).

#### Short and Long Polymorphism Calling

The raw bam files were also processed with Freebayes software (Garrison and Marth, 2012; Figure 1D) to call short polymorphisms. Freebayes is one of the most versatile variants calling software, particularly when the libraries included pool of viral haplotypes as it is in the present study and has been extensively tested and validated on various organisms with different protocols (Olson et al., 2005; Wang et al., 2005). We discarded genomic regions with low complexity (entropy > 1) and regions with a high number of misalignments namely TRL, IRL, IRS, and TRS genomic regions that are duplicated.

Variation of coverage depth and, when available, the orientation of the paired-reads against the reference genome were jointly analyzed to detect SV, deletions, duplications, insertions, inversions, and translocations (Davison et al., 2005). We use Sniffles to perform the SV detection (Figure 1C; Sedlazeck et al., 2018), all the inferred structural variants were checked manually and filtered out as soon as we suspected some alignments artefacts. Most inferred deletions were visually checked with IGV (Thorvaldsdóttir et al., 2013), the remaining types of SV (insertions, duplications, inversions, and translocations) were rare, difficult to check manually and consequently we did not consider those in the rest of the paper. Information from both VCF files, short and long polymorphisms, were merged using vcflib (Garrison, 2019).

#### Consensus Sequence Reconstruction

For each sample, we reconstructed a “consensus sequence” based on the most frequent alleles for SNPs and short insertions/deletions. Into this consensus, we also integrated all long deletions found in the SV calling step, only all “large deletions” inferred by Sniffles and visually checked (Figure 1C, Supplementary Table 1, and Supplementary Figure 1). To create these consensus sequences, we use a combination of bcftools (Li et al., 2009; Li, 2011) and bedtools (Quinlan and Hall, 2010; Figure 1F).

#### Phylogenomic Analysis of Consensus Sequence

We choose to work on the full-length genome (Figures 1C,D,F,G) because among the Malacoherpesviridae family most of the transcripts and proteins have no functional annotation.

All consensus sequences for each samples, plus the publicly available full length OsHV-1 genomes as of March 2021 (MF509813.1, NC_005881.2, KY242785.1, and KY271630.1) and the genome of the acute viral necrotic virus (AVNV) GQ153938.1 were merged and aligned with MAFFT (Katoh and Standley, 2013; Figure 1G). For the multiple sequence alignment, we did not perform any trimming or masking steps as recommended in Tan et al. (2015).

We used a general time reversible evolutionary model with a gamma distribution of the rate of variation among sites and a proportion of invariable sites (GTR + I + G) considered most appropriate according to jModelTest v2.1.10 (Posada, 2008) and based on the Akaike Information Criterion corrected for small sample size (AICc).

We assessed whether there is sufficient temporal signal in our data to proceed with phylogenetic molecular clock analysis and checked there were no sequences whose genetic divergence and sampling date were incongruent with TempEst v1.5.3 (Rambaut et al., 2016) from a maximum likelihood tree build using PhyML v3.1 (Guindon et al., 2010). Time-scaled Bayesian phylogenetic trees were estimated in BEAST v1.10.4 (Suchard et al., 2018) using a GTR + I + G substitution model under a relaxed molecular clock with a lognormal distribution of rates (Figure 1I). A Gaussian Markov Random Field Bayesian skyride coalescent model was used as the tree prior (Minin et al., 2008). A Monte Carlo Markov chain (MCMC) was run for the number of generations needed to achieve stationary distributions (200,000,000 generations) and sampling frequency adjusted accordingly to yield 10,000 samples from the posterior. We used Tracer v1.6 (Rambaut et al., 2016) to visualize the posterior distribution of each parameter and to obtain an estimate of the effective sample size (ESS) after removal of the initial 10% burn-in. We assumed the run had reached sufficient mixing as the ESS of all the parameters was above 200. A maximum clade credibility (MCC) tree was produced after removal of the initial 10% burn-in based on common ancestor heights method (Heled and Bouckaert, 2013) using the auxiliary program TreeAnnotator included in the BEAST package.

#### Statistical Analysis

To test for the correlation between polymorphism and time, host species and stage, we calculated a mean frequency of the targeted polymorphism type (snp, deletion, and standardized on 1,000 bp) across all genes for each individual (to avoid pseudo-replication). We then used a generalized linear model with fixed factors with the following syntax (we add into brackets information about variable nature and modalities numbers).

Polymorphism (SNP or deletion) ∼ sampling date [continuous variable] + host species [factor with two modalities *C*. *gigas*, *O*. *edulis*] + host stage [factor with three modalities: juvenile, pool_larvae, and adult]. All statistics were conducted with R 3.4 (R Development Core Team, 2008) using the linear model, built in “lm” function.

## Results

### Data Quality Observations

As expected, the sequencing depth and the viral genome coverage was correlated to the total number of reads for the different libraries (data not shown). However, the sequencing of fresh moribund individuals, or even better, a pool of infected larvae (as the cost of losing information about the within-host dynamic of viral haplotypes) improves coverage and depth of sequencing. A pragmatic rule of thumb in order to have meaningful and sufficient reads from the virus genome, when sequencing an infected individual host, is to choose a fresh moribund oyster (i.e., an adult, a juvenile or a pool of larvae) harboring more than 5.0 × 10^6^ OsHV-1 copies/mg of fresh flesh and sequence it with a yield of at least 10 × 10^6^ reads per sample (Supplementary Figure 1). This procedure enabled a mean coverage depth of 100X of the whole viral genome for the following samples: LI, NZW17, NZW18, SW6, VIV46-2-m, VIV47-3-m, VIV48-4-m, VIV49-5-m88, VIV56-10-m, and VIV58-12-m. However, it should be kept in mind that most of the reads from a library are from the host’s genome (approximately 95–99%, although this could be as low as 70% in case of pooled infected larvae).

### Viral Haplotype Diversity

**FIGURE 2:**
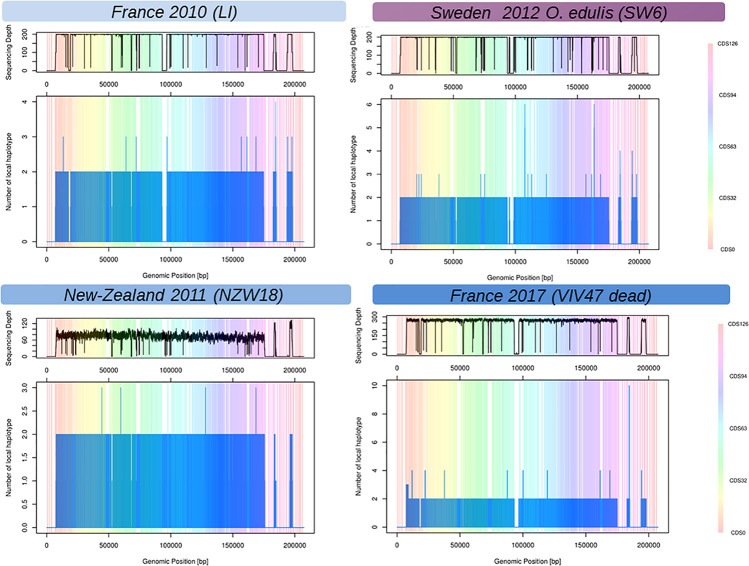
Reconstructed local viral haplotypes for four samples using Shorah with amplicon mode (Zagordi et al., 2011). The X-axis represents the genomic position within the reference viral genome (Davison et al., 2005). The Y-axis is the number of reconstructed viral haplotypes on the focal genomic position. The vertical-colored bands represent the span of the coding DNA sequence (CDS) of the reference viral genome (Davison et al., 2005), one color represents one CDS numbered from 1 to 127. The top panels above the plots represent the coverage depth across the viral genome. Low or no sequencing depth on a genomic region could be interpreted as genomic deletion on the considered samples.

The local reconstruction of haplotype diversity gives an estimate of number of viral haplotypes segregating within one isolate (Figure 2, and Supplementary Figure 2) between 1 and 4 haplotypes (on the CDS basis). As expected, libraries sequenced with short reads and single end, namely PR, MV, and NZ, did not bridge different mutations (SNP or indels) and jeopardize the local assembler heuristics to disentangle sequencing error from true mutations. The coverage depth has a deep impact of the number of haplotypes that were reconstructed. For example, all the VIV samples showed a higher number of reconstructed haplotypes mainly due to a good sequencing depth. Indeed, it is hard to compare the number of viral haplotypes between samples from pool of larvae or juvenile and from one individual host. In summary, the number of viral haplotypes that segregate into infected individual is higher than unit except for samples Poole-Harbor United Kingdom and VIV57-11-m, but both samples have a rather low sequencing depth, and the Poole harbor isolate had previously been passaged through an oyster in a controlled environment. The variation of the number of haplotypes across the viral genome is hard to interpret but some regions seem to harbor more haplotypes nearly inverted and repeated regions.

### Deletions, Substitution Rate and Most Recent Common Ancestors

**FIGURE 3:**
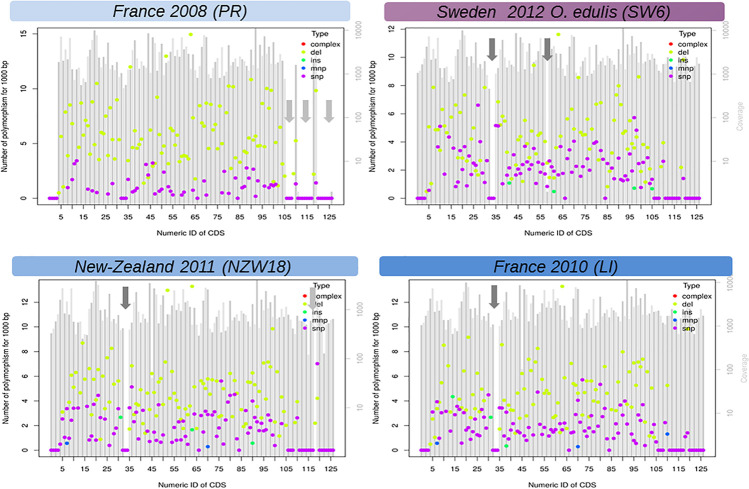
Functional view of DNA variation (mutations) density along the genome of Ostreid herpesvirus 1 (OsHV-1). Here only LI, PR, NZ17, and SW6 are shown. The X-axis is the number of CDS found in the reference annotation genome last accessed (Davison et al., 2005) and the Y-axis is a standardized count of mutations found in 1,000 bp if the SNP density where the same in the targeted CDS. Genomic regions with a low complexity content (repeated sequences) were not considered in this analysis (light gray arrows). SNP, single nucleotide polymorphism; INS, short insertion (<50 bp); DEL, short insertion (<50 bp); MNP, short complex polymorphism, dark gray arrows: large deletion (>50 bp). Figures of all samples (detailed Table 1) are given in Supplementary Files.

The linear regression analysis showed that the accumulation of substitutions is correlated with time and not driven by a hidden species effect (DF = 6/9, *F* = 98.68, *p* value = 1.07 × 10^–7^, time effect estimate = 0.12, time *p* value = 0.023). The greatest modalities of the stage did not have any effect except for the juveniles (or pool of juveniles), however, the unbalanced nature of these factors precludes any serious inferences. In comparison, accumulation of short deletions did not increase with time (model1, DF = 6/9, *F* = 113.8, *p* value = 5.72 × 10^–8^, time effect estimate = 0.02, time *p* value = 0.68). It should be noted, the accumulation of deletions is far above the accumulation of substitutions and seems to be the main process of sequence evolution over time (Figure 3). Our reference mapping approach gives a biased picture of the insertion events into OsHV-1 genome as only sequences which are present in the reference genome can be detected and hence any new sequence inserts larger than read length will be lost during alignment process. Despite this limitation, 89 short insertions could be detected across 22 samples independently of the oyster species and the sampling date. Another important feature of OsHV-1 genome evolution is the presence of large deletions (Figure 3, Supplementary Table 1, and Supplementary Figure 4). The closest sample (PR) in time (and region) compare to OsHV-1 reference genome (Davison et al., 2005) shows no deletion whereas the sample taken from *O*. *edulis* in 2012 showed four large deletions (>>100 bp). A careful visual examination of reads alignment (Figure 3 and Supplementary Figure 1) reveals numerous abrupt drop sequencing depths associated with clipped reads in the CIGAR motif. Both local sequencing depth variation and read clipping were used to infer large deletions and other structural variations (Supplementary Table 1). These large deletions do not seem to be random across the genome, which means that between isolates, deletions did not occur exactly at the same position but are located in the same genomic regions (Supplementary Figure 3, vertical reddish bands).

**FIGURE 4:**
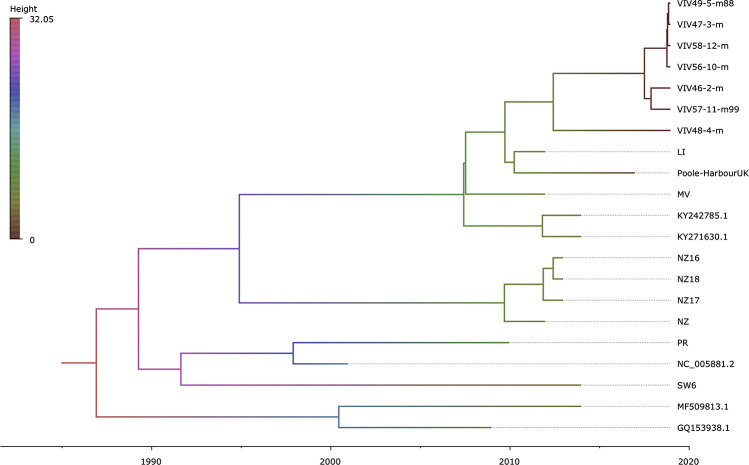
Time-scaled phylogenetic tree of OsHV-1 and chlamys acute necrotic samples. Maximum clade credibility time-calibrated phylogenetic tree generated in BEAST. The tree is scaled in years, with the final sampling year being 2017. The branches are colored accordingly to their mean height across the posterior. This figure was produced with FigTree v1.4.3 (Rambaut, 2019).

Regression of root-to-tip genetic distance against sampling time (Supplementary Figure 5) exposed sufficient temporal signal to proceed with phylogenetic molecular clock analyses. BEAST (Drummond et al., 2012) estimate of the coefficient of variation (CoV) was of 0.701 (95% HPD interval [0.4078, 1.0581]), indicating that strict clock-like evolution could be rejected and validating our choice to use a relaxed molecular clock. OsHV-1 viruses exhibited a mean evolutionary rate of 6.787E-05 nucleotide substitutions per site per year (95% HPD interval [3.5172E-05, 1.0335E-04]). The results indicate that the present genetic diversity seen in OsHV-1 isolates has arisen within the past three decades (Figure 4). Both herpesviruses found in *C*. *farreri* (GQ153938.1) and *A*. *broughtonii* (MF509813.1) group together (Ren et al., 2013; Xia et al., 2015). Their most recent common ancestor (MRCA) is very recent (approximatively 2001) and both species live in sympatry. The virus detected in *O*. *edulis* (SW6) is close to the reference genome (NC_005881.2) and from the OsHV-1 isolate collected from France in 2008 (PR) (Davison et al., 2005). PR and SW6 have their MRCA dated from 1993. Within the *C*. *gigas* host cluster, viral isolates cluster by provenance and sampling year. For example, all isolates from New-Zealand collected in 2010–2011 group together and appear distinct from isolates collected in Europe in 2010, 2015, and 2017 (MV, LI, Poole-Harbour United Kingdom, and all VIV samples). OsHV-1 μVars cluster is represented by the LI and MV isolates from France in 2010 and by both μVar variant A and B (KY242785.1 and KY271630.1) that were isolated from France and Ireland between 2010 and 2014 (Burioli et al., 2017). As expected, all samples from the same epidemic event in 2017 clustered together.

## Discussion

### Summary of the Main Findings

This study brings new insights about the diversity of OsHV-1 at the genomic scale. We show that the OsHV-1 genome has accumulated a large number of mutations since the publication of the reference genome in 2005 obtained from an infected oyster sampled in 1999. Deletions dominate the mutational landscape at the genome level, but our mapping reference approach is much powerful to detect deletions rather than insertions. Our phylogenetic analysis reveals that OsHV-1 diversity is structured by the host species (pectinids, clams, and oyster species), the geographical origin (Europe vs. Oceania) and most importantly the sampling date.

### OsHV-1, a Measurably Evolving Pathogen

Examining the correlation between root-to-tip genetic distance and time suggests OsHV-1 is a measurably evolving pathogen when working at with whole-genome sequencing. The inferred mean evolutionary rate of 6.787E-05 nucleotide substitutions per site per year of OsHV-1 was higher than would be expected under the theory of co-evolution with host species. Potential biases inherent to the analysis conducted have been pointed out (Ho et al., 2005; Emerson, 2007; Debruyne and Poinar, 2009). In particular, it has been demonstrated that molecular rates are accelerated at short timescales on population level studies (Ho, 2005). The most likely explanation for this phenomenon is the persistence of slightly deleterious mutations, which, through purifying selection, would have been eliminated over long timescales (Ho et al., 2005, 2007a,2007b; Ho and Larson, 2006). While conducting selective pressure analyses on a bigger sampling would allow to test that our results were not a consequence of the time dependency of the molecular clock due to short time range of our dataset (i.e., >20 years), our findings are consistent with studies supporting that the evolutionary rates of some dsDNA viruses are comparable to those of RNA and single strand DNA viruses (Hughes et al., 2010; Patrício et al., 2012). Although we acknowledge current limitations of our study, mainly due to the small sample size available, our results demonstrate the feasibility of applying population genetic theory tools such as phylodynamics to a larger and well-designed sampling including virus from healthy animals (Burioli et al., 2016) of whole genome sequences of OsHV-1 and other herpesviruses.

### OsHV-1 Within Host Diversity: Methodological Challenges and Biological Explanations

Viral diversity within infected individuals host is still hard to characterize because of two main biological viral features (Baaijens et al., 2017): (i) the abundance of variants is unknown and (ii) the great diversity and related high mutation rate could challenge classical tools used in bioinformatics (variant calling operations). This problem is even more acute when the abundance of variants is close to the sequencing error rate. Traditionally, in malacoherpesvirus literature, most of the studies have used *de novo* assembly methods to unravel viral diversity (Davison et al., 2005; Ren et al., 2013; Burioli et al., 2017; Bai et al., 2019a). However, at January 2018, most of the available short read assembly methods don’t manage the mixture of haplotypes well, as they are not able to use the molecular phasing information from the reads (Baaijens et al., 2017). Indeed, we choose a pure mapping reference approach to describe viral diversity coupled with local *de novo* assembly. This process allows us to uncover the viral diversity within host. Our estimates about the true number of viral haplotypes within infected individuals were very conservative (lower bound) since we are not able to phase variants above 150 bp. To explain the source of the diversity within an infected individual, several non-exclusives hypotheses could be formulated: First, diversity within host is due to a multiple infection by several viral lineages. This means that several OsHV-1 haplotypes could be found in sea water during mortality outbreak. However, viral particles in sea water are hard to detect even when using passive sensor systems [lab detection of virus in seawater (Vincent-Hubert et al., 2017) and Hubert in prep (*in situ* detection of virus in sea water)]. Some alternatives approaches using ultra-filtration of large sea water volume are a promising way to check this hypothesis and we would help in finding if various haplotypes are present at the onset of the disease. Again, using ultra-filtration methods of sea water, we could expect an increase of the number of variants during the spread of the disease through a population. Such hypothesis could also be tested throughout the genomic relatedness among viral variants within individuals and variants from the previous years. The last hypothesis concerns *de novo* mutations during the infection process, viruses use host cellular machinery to replicate, there are several mechanisms which could give arise to *de novo* variants mediated by host antiviral defense systems (Sanjuán and Domingo-Calap, 2016).

Recently, extensive anti-viral mechanisms were characterized; Rosani et al. (2019) demonstrated that Adenosine deaminase enzymes of the ADAR family are found in the Pacific oyster and have the ability to post-transcriptionally modify OsHV-1 RNA in the form of A-to-I conversions. Indeed, investigators showed that viral genome have evolved to reduce the number of deamination targets along most of OsHV-1 CDS. The life cycle of herpes virus is also prone to mutation via recombination mechanisms which have been demonstrated in other herpesvirus or close-related as cytomegalovirus in human (Wilkinson and Weller, 2003; Norberg et al., 2007; Sijmons et al., 2015; Balloux et al., 2016; Kolb et al., 2017; Tomer et al., 2019). Although two DNA Recombination-Initiating Promoter Motifs have been reported as enriched among Malacoherpesviridae (Rosani and Venier, 2017), the evidence of such mechanisms is yet to be characterized in Malacoherpesviridae. The evolutionary advantage of having multiple viral haplotypes within one infected individual is of prime interest, since lethal or sub-lethal viral haplotypes could be maintained in the population thank to genetic complementation and be an additional reservoir of standing genetic variation (Segredo-Otero and Sanjuán, 2019).

### Large Genomic Deletions Are a Good Markers of Virus Origin

In this study, we were able to compare the genomic structure of various viral isolates from several host species. All isolates were compared to the oldest reference genome published (Davison et al., 2005) which is likely to be the ancestor of most of our samples. Reads from all the samples were mapped to the Davison reference genome, however, visual inspection of sequencing depth and structural variant analysis reveals tremendous genetic variation between virus isolates. Notably virus isolates from *O*. *edulis* have two specific deletions in ORF101 (48 bp deleted) and ORF105 (49 bp deleted) that are not shared with the other isolates. We hypothesize that this particular haplotype who is attached to *O*. *edulis* (named OsHV-1 SW6) is a virus strain “adapted” to infect this species. If our expectation is true, this haplotype will be found in other infected *O*. *edulis* population. In addition, isolates from New-Zealand (NZ16-NZ17-NZ18) share only two of the three observed larges deletions identified in France isolates (LI) probably because the New-Zealand samples are from a different virus reservoir. The exact molecular mechanism(s) of such deletion(s) are unknown, but they are not deleterious for the viral replication as they are maintained in the actual viral population. In addition, deletions in the OsHV-1 are not completely random across the samples they tend to occur in the regions 17,700–19,000 bp (ORF11), 52,000–52,800 bp (ORF35, ORF36, and ORF37), 67,972–68,573 bp (mainly ORF48), 93,000–98,500 bp (ORF62, ORF63, and ORF64). Burioli et al. (2017) observed the same deletions as we observe in the LI and VIV samples. The scarcities of annotation in these regions do not help us to formulate a functional hypothesis.

### Refining Malacoherpiviradae Classification to Get a Better Understanding of Viral Micro Evolution

Ostreid herpesvirus 1 is usually described as a generalist herpesvirus that is able to infect various marine bivalves species across the world (Arzul et al., 2001, 2017; Davison et al., 2005; Gao et al., 2018; Kim et al., 2019). However, our phylogenetic analysis shows that such view is probably too simple and more elaborate hypothesis are needed. Recently, OsHV-1 found on two other bivalve species (*C*. *farreri* and *A*. *broughtonii* mainly in China) (Xia et al., 2015; Bai et al., 2017, 2018, 2019b) seems to have a recent common ancestor, the MRCA is dated from 1991 to 2006. In addition, there is a growing number of report of OsHV-1 DNA in novel host species *Scapharca subcrenata* and *Agropecten irridians* (Gao et al., 2018; Kim et al., 2019). In addition, we also observed a unique type of deletion in this isolate (Figure 3, SW6 sample) compare to other isolates from *C*. *gigas*. All these inferred host shifts could be explained by two hypotheses: (1) We are revealing an old viral diversity, i.e., various viral haplotypes are adapted to various host species, but this diversity was not accessible to previous studies because of technological limitations (MLST, gene concatenation, microsatellite, and Sanger sequencing). However, this hypothesis does explain why MRCA are so recent (less than 50 years). (2) We are observing an ongoing specialization (speciation) of a generalist virus [OsHV-1 census (Davison et al., 2005)] to a specialized viral populations OsHV-1-SB on *A*. *broughtonii*, AVNV on *C*. *farreri*, OsHV-1 Var on *P*. *maximus*, OsHV-1 μVars on *C*. *gigas*, and now OsHV-1 SW6 on *O*. *edulis*, a mechanism possibly supported by the different host antiviral responses. For the later haplotype, we hypothesize a host shift from *C*. *gigas* to *O*. *edulis* and the estimated date of this shift (1992) which is congruent with the history of the introduction of *C*. *gigas* in Scandinavia (Wrange et al., 2010; Mortensen et al., 2016; Anglès d’Auriac et al., 2017). These recent host shifts could be explained by two mechanisms well documented in other farming animal and plants (Jones et al., 2013; Bai et al., 2015; Mineur et al., 2015). The first mechanism is the introduction of *C*. *gigas* (and OsHV-1) world-wide for its extraordinary adaptive capacity and the second is an intensification of *C*. *gigas* farming (imported spat, spat from hatcheries, high field density, triploid’s oyster, and extension of field surfaces). Indeed, OsHV-1 could reach quite high number of genome copies per infection events since the cultivated density of susceptible animals are much higher than natural beds, leading to an increase of contact with a larger number of bivalve’s taxa than ever. Host shift is likely to be just a matter of time because of the abundant genomic viral diversity. We postulate that OsHV-1 taxon is under specialization in novel populations and/or bivalve species due to world-wide expansion of *C*. *gigas* shellfish farming in the 1970s.

In order to discriminate between the first and the second hypothesis regarding variants, we need more detailed study of isolates from various bivalve species, most notably including old and novel sympatric species of *C*. *gigas*. In fact, we need a better understanding of how OsHV-1 is constrained, or not, to a specific host species and how frequently it could jump from one species to another species (Brito et al., 2021). Only well-replicated phylogenetic studies encompassing the whole viral genome information will help-us to better understand OsHV-1 ecology and evolution.

### Conclusion and Perspectives

We have characterized the viral genomic diversity within an infected individual; our estimates are probably lower bounds of the exact number of haplotypes. To refine and notably to understand complementation between haplotypes within individuals new techniques, such as long read sequencing, will be required (Bai et al., 2019a). The observed viral diversity is generally well structured according to host species, then geographical origins and finally sampling date. There is an urgent need to assess the effect of some mutations on viral phenotype namely pathogenicity (virulence and infectivity) for a given host species and populations, including basic information about which viral haplotype can infect which host species. These crucial questions about virus ecology should be explored to be able to understand the potential of OsHV-1 to jump from one species to the next.

This study thus lays the groundwork for future studies to further analyze the relationships between viral diversity and pathogenicity across bivalve species and strains.

## Data Availability Statement

The data presented in the study are deposited in the NCBI Sequence Read Archive (SRA) under repository, accession number of the BioProject ID: PRJNA721248.

## Author Contributions

This study is the result of a collective work. BM, J-BL, J-FP, and TR conceived this study, and participated in its design. SH and CP performed the sample preparation for Illumina sequencing. J-BL, AB, MJ, and BM performed genomic analyses. BM, MJ, and J-BL interpreted the results and drafted the manuscript. All authors read, corrected, and approved the final manuscript.

## Conflict of Interest

The authors declare that the research was conducted in the absence of any commercial or financial relationships that could be construed as a potential conflict of interest.

## Supplementary Material

Supplementary Figure 1 Correlation between the assessed viral copy numbers per μL per ng of gills from infected individuals for all the individuals from VIV batches and the sequencing depth of each sample. The labels of the sample should be interpreted as the family name (i.e., viv57) followed by the individual number (viv57-11) and the final status at the end of the experimental infection “v” stand for alive (viv57-11-v3) and “m” for dead (viv57-11-m99).

Supplementary Figure 2 Reconstructed local viral haplotypes for four samples using Haploclique (Töpfer et al., 2013). The X-axis represents a genomic position within OsHV-1 reference genome in base pair; the Y-axis is the number of haplotypes reconstituted at the focal genomic position. Large deletions are visible in the sequencing top panels and in the number of reconstructed haplotypes.

Supplementary Figure 3 Local viral haplotype reconstruction using QuasiRecomb software on genomic regions without any abrupt drop of sequencing depth (large deletions). Reconstructions have been done on CDS basis (using the reference genome annotation from Davison et al. (2005). The X-axis represents the genomic position within the reference viral genome. The Y-axis is the number of quasispecies. The black number corresponding to ORF. In red, we represented the larges deletion found in the consensus viral genome of each isolates, orange illustrate genomic regions where the alignment was not good enough to allow haplotype reconstruction. Large deletions were detected after visual inspection of bam file and sniffles detection.

Supplementary Figure 4 Illustration of the variety of polymorphism found in OsHV-1 genome mapped against the reference genome from Davison et al. (2005). Some visual patterns observed in Bam files motivate the search of viral haplotypes. Here both Bam and Vcf files are viewed using IGV (Thorvaldsdóttir et al., 2013). The blue arrowed highlights some detected polymorphisms and red arrows point out undetected potential variants. Horizontal arrows showed the large deletions, i.e., structural variations, and vertical arrows pointed out short polymorphism, i.e., short indel and SNPs. Each picture (one per sample) is composed of a bandplot showing the sequencing depth (top panel) of the considered genomic regions followed by the alignment of individual reads (bottom panel). The sequencing depth is between 0 and 4,000 X for the LI and SW6 samples, and 0–190 for NZ17 samples. **(A)** The observed region is from 94,000 to 100,000 bp on LI, NC17, and SW6 isolates. **(B)** The observed region is from 71,400 to 73,000 bp. **(C)** Zoom in the 77,900–79,000 bp in the NZ17 samples, it seems that there is a physical linkage between a short deletion and a SNP leading to conclusion that there are at least two haplotypes segregating in this region. **(D)** Zoom in the 21,700–22,800 bp region of the SW6 samples. The extremely large sequence depth of this sample allows to infer that few haplotypes segregating in this population show duplication.

Supplementary Figure 5 Clock-likeness of OsHV-1 genomes. Regression of root-to-tip genetic distance against sampling time were computed using TempEst v1.5.3 (Tan et al., 2015) using two rooting methods (best fit in TempEst and midpoint). Sufficient and robust temporal signal was observed to proceed with phylogenetic molecular clock analyses.
