# Supplementary figures and images for: Genomic Diversity of the Ostreid Herpesvirus Type 1 Across Time and Location and Among Host Species
Source: Front Microbiol. 2021 Jul 13;12:711377. doi: 10.3389/fmicb.2021.711377 (PMC8313985; doi:10.3389/fmicb.2021.711377)

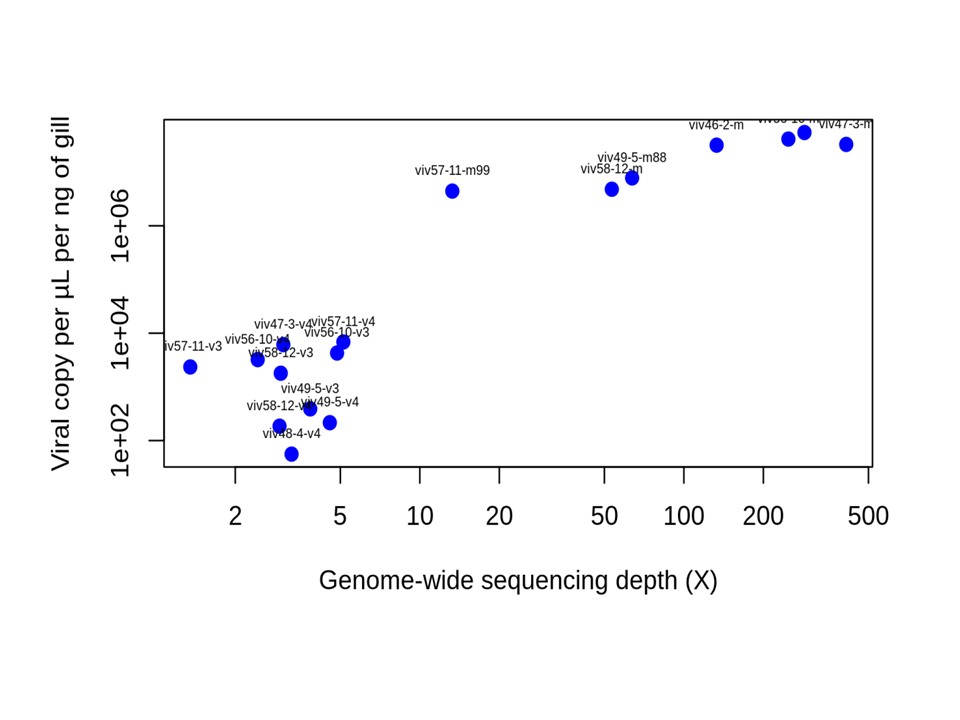

Supplement: Supplementary Figure 1 — Correlation between the assessed viral copy numbers per μL per ng of gills from infected individuals for all the individuals from VIV batches and the sequencing depth of each sample. The labels of the sample should be interpreted as the family name (i.e., viv57) followed by the individual number (viv57-11) and the final status at the end of the experimental infection “v” stand for alive (viv57-11-v3) and “m” for dead (viv57-11-m99). [file Image_1.TIF]

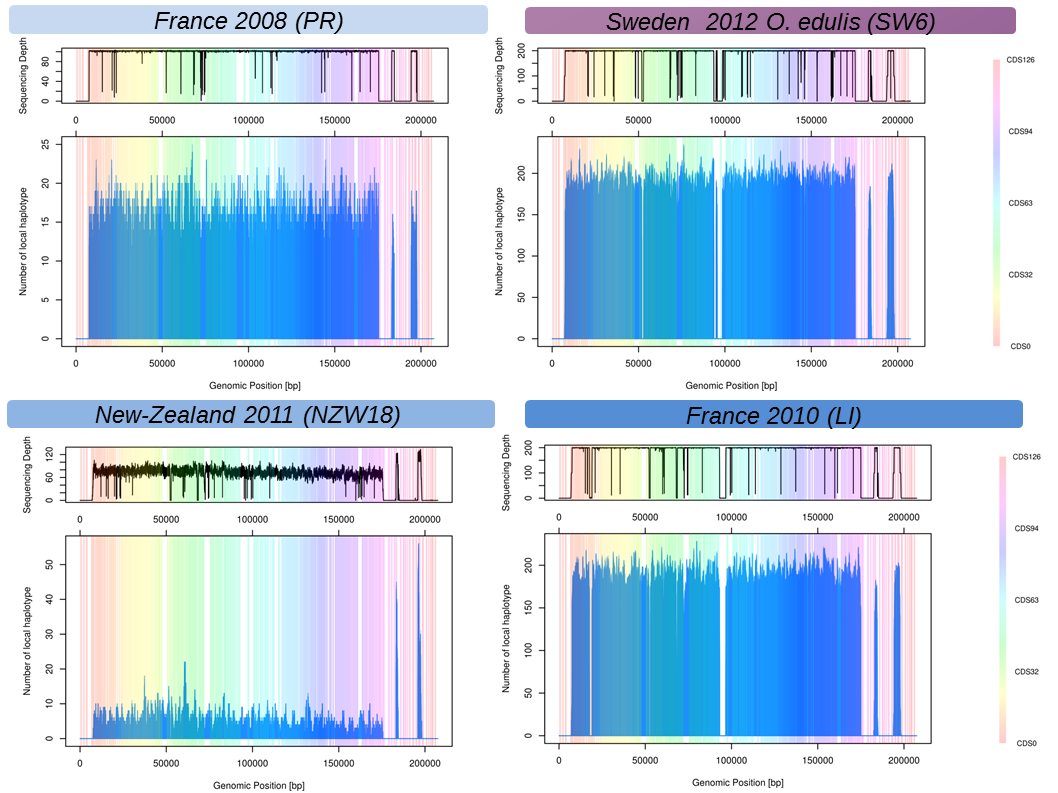

Supplement: Supplementary Figure 2 — Reconstructed local viral haplotypes for four samples using Haploclique (Töpfer et al., 2013). The X-axis represents a genomic position within OsHV-1 reference genome in base pair; the Y-axis is the number of haplotypes reconstituted at the focal genomic position. Large deletions are visible in the sequencing top panels and in the number of reconstructed haplotypes. [file Image_2.TIF]

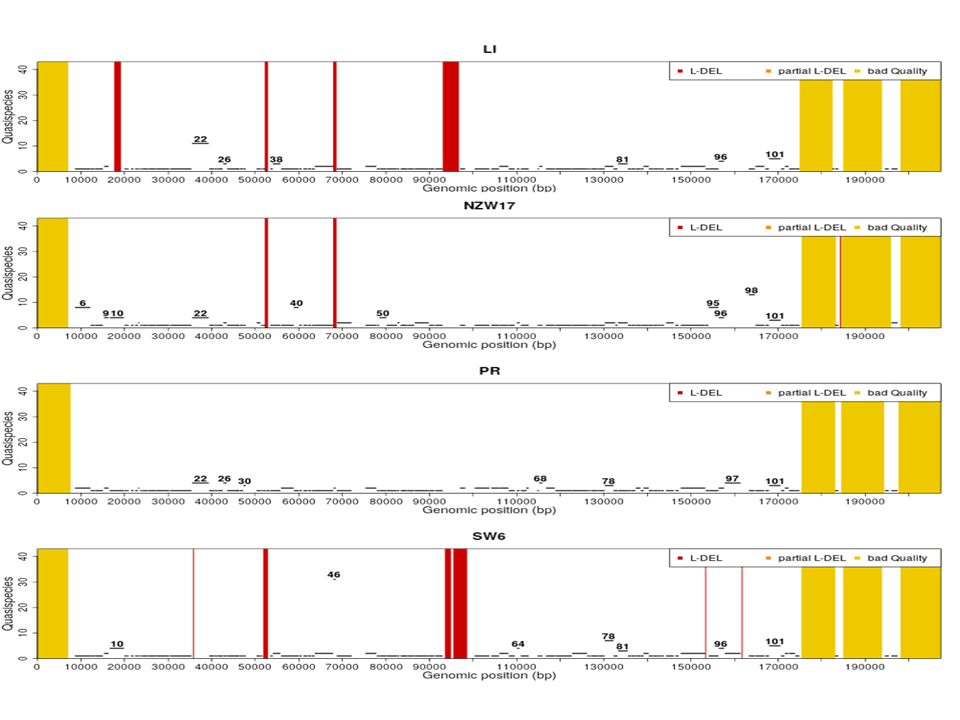

Supplement: Supplementary Figure 3 — Local viral haplotype reconstruction using QuasiRecomb software on genomic regions without any abrupt drop of sequencing depth (large deletions). Reconstructions have been done on CDS basis (using the reference genome annotation from Davison et al. (2005). The X-axis represents the genomic position within the reference viral genome. The Y-axis is the number of quasispecies. The black number corresponding to ORF. In red, we represented the larges deletion found in the consensus viral genome of each isolates, orange illustrate genomic regions where the alignment was not good enough to allow haplotype reconstruction. Large deletions were detected after visual inspection of bam file and sniffles detection. [file Image_3.TIF]

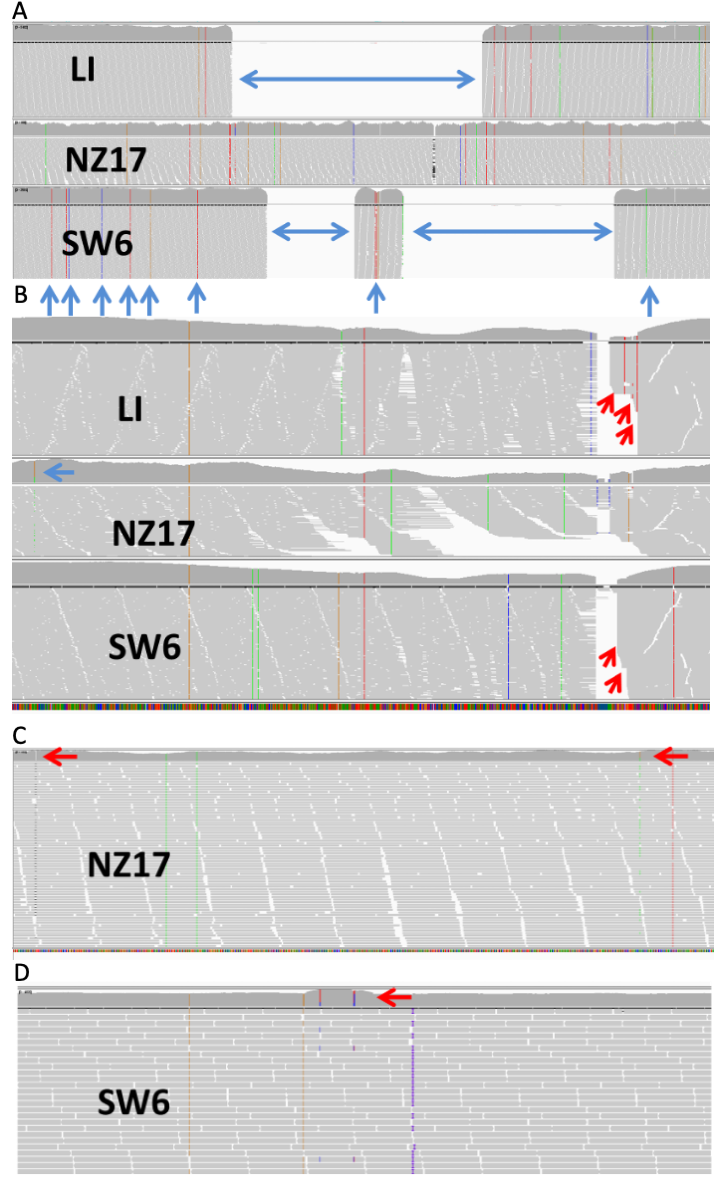

Supplement: Supplementary Figure 4 — Illustration of the variety of polymorphism found in OsHV-1 genome mapped against the reference genome from Davison et al. (2005). Some visual patterns observed in Bam files motivate the search of viral haplotypes. Here both Bam and Vcf files are viewed using IGV (Thorvaldsdóttir et al., 2013). The blue arrowed highlights some detected polymorphisms and red arrows point out undetected potential variants. Horizontal arrows showed the large deletions, i.e., structural variations, and vertical arrows pointed out short polymorphism, i.e., short indel and SNPs. Each picture (one per sample) is composed of a bandplot showing the sequencing depth (top panel) of the considered genomic regions followed by the alignment of individual reads (bottom panel). The sequencing depth is between 0 and 4,000 X for the LI and SW6 samples, and 0–190 for NZ17 samples. (A) The observed region is from 94,000 to 100,000 bp on LI, NC17, and SW6 isolates. (B) The observed region is from 71,400 to 73,000 bp. (C) Zoom in the 77,900–79,000 bp in the NZ17 samples, it seems that there is a physical linkage between a short deletion and a SNP leading to conclusion that there are at least two haplotypes segregating in this region. (D) Zoom in the 21,700–22,800 bp region of the SW6 samples. The extremely large sequence depth of this sample allows to infer that few haplotypes segregating in this population show duplication. [file Image_4.TIFF]

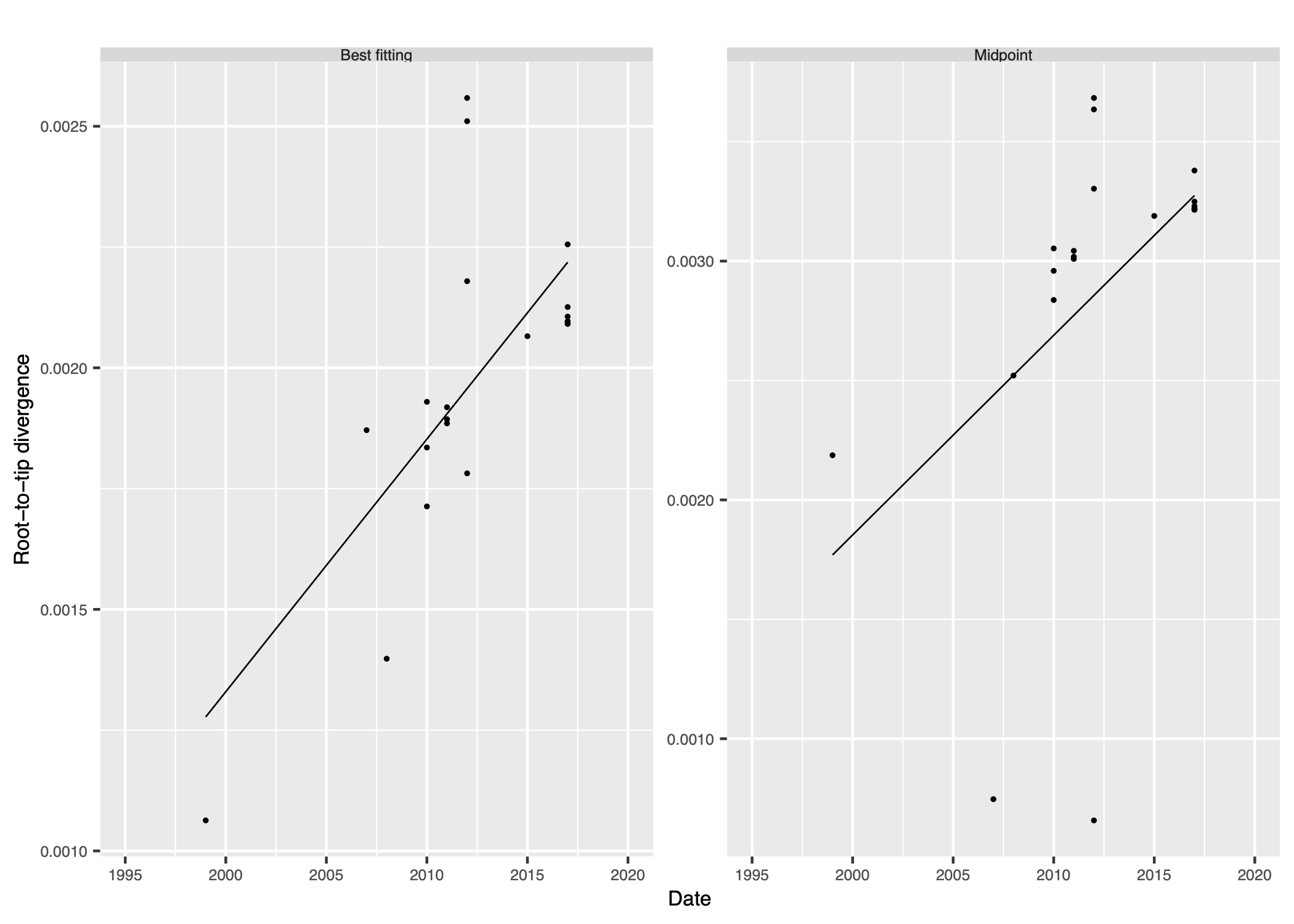

Supplement: Supplementary Figure 5 — Clock-likeness of OsHV-1 genomes. Regression of root-to-tip genetic distance against sampling time were computed using TempEst v1.5.3 (Tan et al., 2015) using two rooting methods (best fit in TempEst and midpoint). Sufficient and robust temporal signal was observed to proceed with phylogenetic molecular clock analyses. [file Image_5.TIFF]
