# Supplementary material for: Genomic Diversity of the Ostreid Herpesvirus Type 1 Across Time and Location and Among Host Species
Source: Front Microbiol. 2021 Jul 13;12:711377. doi: 10.3389/fmicb.2021.711377 (PMC8313985; doi:10.3389/fmicb.2021.711377)
Supplement: Supplementary file 6 [file Table_1.docx]

Table S1: List of detected structural variation in OsHV-1 genome for each given sample (deletion, inversion or duplication superior to 100 bp of length). Some structural variations are at the heterozygous states (with more than two alleles, Figure 2). The analysis was done with Sniffles (43).

| Sample’s ID | Large inversion | Large deletion | Large duplication |
| --- | --- | --- | --- |
| LI | 1* | 2 | 1* |
| NZW16 | 0 | 4 | 0 |
| NZW17 | 0 | 4 | 0 |
| NZW18 | 0 | 4 | 0 |
| Poole-HarbourUK | 0 | 4 | 0 |
| PR (same as *Davison et al, 2005*) | 0 | 0 | 0 |
| SW6 | 0 | 5 | 0 |
| VIV46-2-m | 0 | 1 | 0 |
| VIV47-3-m | 0 | 5 | 0 |
| VIV48-4-m | 0 | 4 | 0 |
| VIV49-5-m88 | 0 | 1 | 0 |
| VIV56-10-m | 0 | 5 | 0 |
| VIV57-11-m99 | 0 | 4 | 0 |
| VIV58-12-m | 0 | 4 | 0 |

* Structural variations that were not validated after visual inspections of the bam files.
